# Implementing Low-Cost, Community-Based Exercise Programs for Middle-Aged and Older Patients with Type 2 Diabetes: What Are the Benefits for Glycemic Control and Cardiovascular Risk?

**DOI:** 10.3390/ijerph14091057

**Published:** 2017-09-13

**Authors:** Romeu Mendes, Nelson Sousa, Victor Machado Reis, Jose Luis Themudo-Barata

**Affiliations:** 1 Public Health Unit, ACES Douro I-Marão e Douro Norte, Northern Region Health Administration, 5000-524 Vila Real, Portugal; 2 Department of Sport Sciences, Exercise and Health, University of Trás-os-Montes e Alto Douro, 5000-801 Vila Real, Portugal; nelsons@utad.pt (N.S.); vmreis@utad.pt (V.M.R.); 3 EPIUnit-Instituto de Saúde Pública, Universidade do Porto, 4050-600 Porto, Portugal; 4 CIDESD-Research Center in Sports Sciences, Health Sciences and Human Development, 5000-801 Vila Real, Portugal; 5 Faculty of Health Sciences, University of Beira Interior, Cova da Beira Hospital Centre, 6200-506 Covilhã, Portugal; jlthemudobarata@gmail.com

**Keywords:** type 2 diabetes, exercise, physical activity, cardiovascular risk, community-based interventions

## Abstract

Background: The purpose of this study was to analyze the effects of a long-term, community-based, combined exercise program developed with low-cost exercise strategies on glycemic control and cardiovascular risk factors in middle-aged and older patients with type 2 diabetes. Methods: Participants (*n* = 124; 63.25 ± 7.20 years old) engaged in either a 9-month supervised exercise program (*n* = 39; consisting of combined aerobic, resistance, agility/balance, and flexibility exercise; three sessions per week; 70 min per session) or a control group (*n* = 85) who maintained their usual care. Glycemic control, lipid profile, blood pressure, anthropometric profile, and the 10-year risk of coronary artery disease were assessed before and after the 9-month intervention. Results: A significant time * group interaction effect (*p* < 0.001) was identified in the values of the glycated hemoglobin, fasting plasma glucose, total cholesterol, LDL cholesterol, HDL cholesterol, triglycerides, systolic blood pressure, diastolic blood pressure, body mass index, waist circumference, and the 10-year risk of coronary artery disease. Conclusions: A long-term, community-based, combined exercise program developed with low-cost exercise strategies was effective in inducing significant benefits on glycemic control, lipid profile, blood pressure, anthropometric profile, and the 10-year risk of coronary artery disease in middle-aged and older patients with type 2 diabetes. Clinical Trial Identification Number: ISRCTN09240628.

## 1. Introduction

Diabetes was responsible for approximately 5 million deaths worldwide in 2015, representing 14.5% of global, all-cause mortality in people aged 20 to 79 years [1]. Cardiovascular diseases are the leading cause of death and an important cause of morbidity among persons with diabetes, especially due to the increased incidence of coronary artery disease and strokes [2,3].

Physical activity is considered one of the cornerstones of type 2 diabetes (T2D) treatment and control as it is associated with a decreased incidence of cardiovascular disease and a lower all-cause mortality [4,5]. Exercise (physical activity that is planned and structured) seems to promote additional benefits on glycemic control and cardiovascular risk factors compared to daily life physical activity [6,7].

The effects of regular exercise programs developed according to international exercise recommendations (combined aerobic and resistance exercise) [7,8] for patients with T2D on glycemic control and cardiovascular risk factors are very well established [9,10]. However, the majority of intervention studies have developed exercise programs with expensive equipment such as ergometers for aerobic exercise (treadmills, stationary bikes, rowing machines, steppers, and ellipticals) and resistance machines for resistance exercise. Accessing these material resources represents an elevated economic cost in a population already burdened with high health expenditures due to diabetes and related comorbidities treatments. Therefore, these type of facilities are not accessible to most of patients with T2D, especially in community settings such as in primary health care and elderly institutions [11,12].

Community-based exercise programs for groups of individuals with homogeneous characteristics, such as specific age-groups or chronic diseases, are recommended interventions by the World Health Organization [13] to promote physical activity. These types of community interventions seem to have a greater applicability than interventions at the individual level, and are associated with greater long-term compliance in patients with T2D. However these programs typically fail to meet the international physical activity guidelines and/or to use low-cost exercise strategies [14,15].

In order to allocate resources to physical activity interventions, decision-makers need to be informed about the efficacy of these programs in changing health outcomes, especially in regards to diabetes control and decreasing cardiovascular risk.

The published literature still does not answer the question whether these low-cost physical activity interventions are effective at controlling the aforementioned health conditions.

This study aimed to analyze the effects of a long-term, community-based combined exercise program developed with low-cost exercise strategies on glycemic control and major modifiable cardiovascular risk factors in middle-aged and older patients with T2D.

## 2. Materials and Methods

### 2.1. Study Design

This was a non-randomized controlled study. Participants were engaged in a 9-month supervised exercise program (EXE) or a control group (CON). Glycemic control (glycated hemoglobin [HbA1c] and fasting plasma glucose [FPG]), lipid profile (total cholesterol, LDL cholesterol, HDL cholesterol, and triglycerides), blood pressure (systolic blood pressure [SBP] and diastolic blood pressure [DBP]), anthropometric profile (body mass index [BMI] and waist circumference) and the 10-year risk of coronary artery disease (CAD) events were assessed as primary outcomes before (baseline) and after the intervention (final). Habitual physical activity (HPA) and dietary pattern were also assessed as secondary outcomes.

### 2.2. Participants

Participants were recruited from a diabetes clinic at a local hospital according to the following inclusion criteria: aged 55 to 75 years; diagnosis of T2D for at least one year; HbA1c less than 10%; pharmacological regimen stabilized for at least three months; major complications of diabetes screened and controlled (diabetic retinopathy, diabetic nephropathy, diabetic foot, and major cardiovascular risk factors); without limitations in gait or balance; independent living in the community; without participation in supervised exercise programs in the last 6 months; non-smokers in the last 6 months; consistent dietary pattern for at least 6 months. Medical Doctors and Nurses identified 170 patients with the eligibility criteria. All agreed to participate in a long term follow-up study. An application for participation in a free of charge, long-term exercise program was opened for these patients. Sixty-eight individuals applied to this program and 60 (30 women and 30 men) were randomly selected to participate (EXE). All other individuals (*n* = 110), independently of having (*n* = 8) or not (*n* = 102) applied to the exercise program, were integrated in the CON.

All participants received instructions to maintain daily-life routines (lifestyle-related physical activity and dietary pattern), and continue with usual care (diabetes consultations at the local hospital and pharmacological regimen) throughout the study’s duration.

Before exercise program engagement, all EXE participants underwent a detailed medical evaluation to screen for relative or absolute contraindications to vigorous intensity exercise, including a maximal treadmill stress test [7,16].

The study’s protocol was approved by the local hospital’s ethics committee in accordance with the Declaration of Helsinki (36/2009). All individuals were informed about the benefits and risks of the research prior to signing an institutionally-approved informed consent document to participate in the study.

### 2.3. Evaluations

Data were collected at the local hospital two weeks before the start of the exercise program (baseline) and two weeks immediately after its end (final).

Glycemic control (HbA1c and FPG) and lipid profile (total cholesterol, LDL cholesterol, HDL cholesterol, and triglycerides) were determined by a fasting (minimum of 8 h) venous blood analysis performed in the local hospital through standard international laboratory methods.

Blood pressure (SBP and DBP) was assessed according to international recommendations [17] and using an automatic digital blood pressure device (BP-8800, Colin Corporation, Komaki, Japan).

BMI was calculated measuring body mass and height (mass [kg]/height [m]^2^) using a digital weight scale (SECA 778, SECA Corporation, Hamburg, Germany) with stadiometer (SECA 220, SECA Corporation, Hamburg, Germany). Waist circumference was measured according to international recommendations [18] using an anthropometric tape (SECA 201, SECA Corporation, Hamburg, Germany).

Ten-year risk of CAD events (fatal and non-fatal) was calculated through the United Kingdom Prospective Diabetes Study Risk Engine version 2.0 [19].

HPA was assessed using the International Physical Activity Questionnaire (IPAQ, short format, self-administered version [20]). Final evaluations only included non-supervised physical activity in order to control lifestyle-related physical activity.

Dietary pattern was analyzed by a dietitian through a 24-h food recall, in order to identify qualitative changes (number of daily meals, proportions, and variety or combinations of different foods and beverages) in the subject’s diet during the intervention period, as well as to identify the usage of food supplements.

### 2.4. Exercise Program

**Figure 1:**
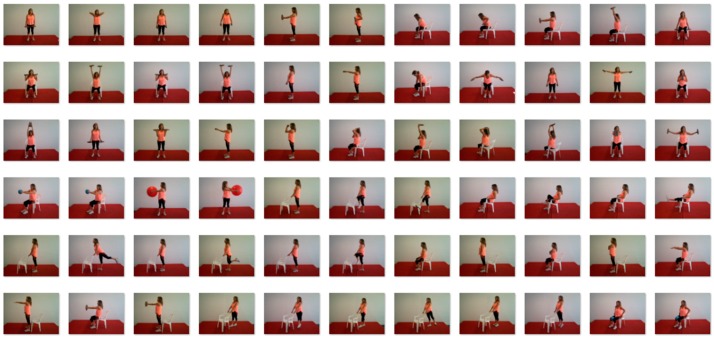
Resistance exercises used in the exercise program.

Participants were engaged in *Diabetes em Movimento* which is a community-based exercise program for patients with T2D developed in Portugal [21]. This exercise program was prepared according to the international exercise recommendations for patients with T2D and also the international recommendations on falls prevention. The program involved the combination of aerobic, resistance, agility/balance, and flexibility exercise within each session [8,22]. The program’s sessions were held three times per week on non-consecutive days over nine months and took place in a municipal sports complex (Covilhã, Portugal) equipped with an all-weather running track, lawns, and an exercise room. Only low-cost materials were used such as chairs, water bottles filled with sand (0.5 L; ±0.75 kg), dumbbells (1, 2 and 3 kg), fitness balls, beacons, stakes, shopping baskets, a volleyball portable kit, and sports vests. Exercise sessions were conducted in groups of 30 participants, supervised by an exercise professional and a nurse (both with specific training on exercise-related injuries and adverse events [16]), and lasted about 70 minutes, according to the following structure:

1. Warm-up (5 min) consisting of continuous brisk walking at the all-weather running track.
2. Aerobic exercise (30 min) at the all-weather running track and lawns, consisting of moderate-continuous brisk walking (12–13 points on Borg’s scale [23]) and high-intensity interval walking (relay-races, walking with external load, obstacles and stairs circuits; 14–17 points on Borg’s scale). The ratio between moderate-continuous and high-intensity interval walking activities was 1:1 min.
3. Resistance exercise for muscle strengthening (20 min) in the exercise room. In each session, six exercises were performed (three for the lower limbs and three for the upper limbs and torso) with bodyweight, chairs, sand bottles, dumbbells, and fitness balls (Figure 1). Exercises were organized in a circuit mode with exercises for lower limbs alternating with exercises for upper limbs and torso. There was no resting period between each exercise, and only a 1-min rest between each circuit. The number of circuits ranged progressively from one (adaptation phase) to four (in the last two months). Twenty repetitions were performed in each bilateral exercise, and 30 repetitions were performed alternately for each limb in unilateral exercises. Exercise load was selected in order to achieve local muscle fatigue during the execution of the last repetitions of each exercise. Load increase was promoted when the last repetitions of each exercise were performed without local muscle fatigue. All exercises were performed simultaneously by all participants, and the movement’s execution time and the rest time were controlled by an exercise professional.
4. Agility/balance exercise (10 min), consisting of small-sided and conditioned team games.
5. Flexibility exercise (5 min) through a sequence of static and dynamic stretches was performed with the support of chairs. Static positions were held for 15 seconds and dynamic stretching was performed for 10 repetitions.

Five different exercise sessions models were prepared. Each of them had different aerobic, resistance, and agility/balance exercises successively applied over time to induce stimuli variability. Exercise sessions were planned to have moderate-to-vigorous intensity (12–17 points on a rate of perceived exertion scale with 6–20 points). Exercise intensity was systematically controlled using Borg’s rate of perceived exertion scale (6–20 points, [23]) and adjusted if necessary during aerobic, resistance, and agility/balance exercise. At the end of each session, all participants were asked to rate the session’s overall intensity. The participant’s attendance, as well as the occurrence of exercise-related injuries and adverse events, was also recorded.

The methods of this exercise program were already tested for their impact on physical fitness (aerobic fitness, muscle strength, agility/balance, and flexibility) in patients with T2D, as previously published by our research team [24]. Moreover, the cost of program implementation was also reported (18.40 €/patient/month; only direct costs, calculated by the health services perspective [25]).

### 2.5. Data Analysis

A per-protocol analysis was used in order to assess the efficacy of the program’s specific methods, since the main goal of this study was to test the effects of the low-cost and high-applicability exercises, specifically on diabetes control and cardiovascular risk.

The following exclusion criteria were established during the study period: dropout of the exercise program; adherence to the exercise sessions <65%; participation in other supervised exercise sessions; changes in pharmacological treatment for T2D, hypertension, or dyslipidemia; changes in dietary pattern; accident, illness or surgery with hospitalization; development of pathology with a limitation on the performance of exercise sessions’ activities; and loss of contact with hospital diabetology consultations.

To analyze the effects of the exercise program on HbA1c, FPG, total cholesterol, LDL cholesterol, HDL cholesterol, triglycerides, SBP, DBP, BMI, waist circumference, and the 10-year risk of CAD events, a Split-Plot analysis of variance (time *group) with repeated measures was performed. Partial Eta^2^ values (η^2^_p_) were reported to quantify the effect sizes. To compare HPA (non-supervised) between EXE and CON in the two evaluation moments, Mann–Whitney U tests for independent samples were performed. The level of statistical significance was set at *p* < 0.05 and data was analyzed with PASW Statistics (version 20, SPSS Inc., Chicago, IL, USA). Data are shown as mean ± standard deviation and as median (interquartile range) for the HPA values. 

## 3. Results

During the study period the following exclusion criteria were observed in the EXE: dropout of the exercise program (*n* = 7 [lack of time, *n* = 2; support to a sick spouse, *n* = 3; emigration, *n* = 2]); adherence to the exercise sessions <65% (*n* = 6); changes in pharmacological treatment for T2D, hypertension, or dyslipidemia (*n* = 4; increase in the dose of medications); changes in dietary pattern (*n* = 1); accident, illness, or surgery with hospitalization (*n* = 2); development of pathology with a limitation on the performance of program activities (*n* = 1).

The following exclusion criteria were observed in the CON: participation in other supervised exercise sessions (*n* = 1); changes in pharmacological treatment for T2D, hypertension, or dyslipidemia (*n* = 13; an increase in the dose of medications); changes in dietary pattern (*n* = 2); accident, illness, or surgery with hospitalization (*n* = 6); and loss of contact with hospital diabetology consultations (*n* = 3).

**Figure 2:**
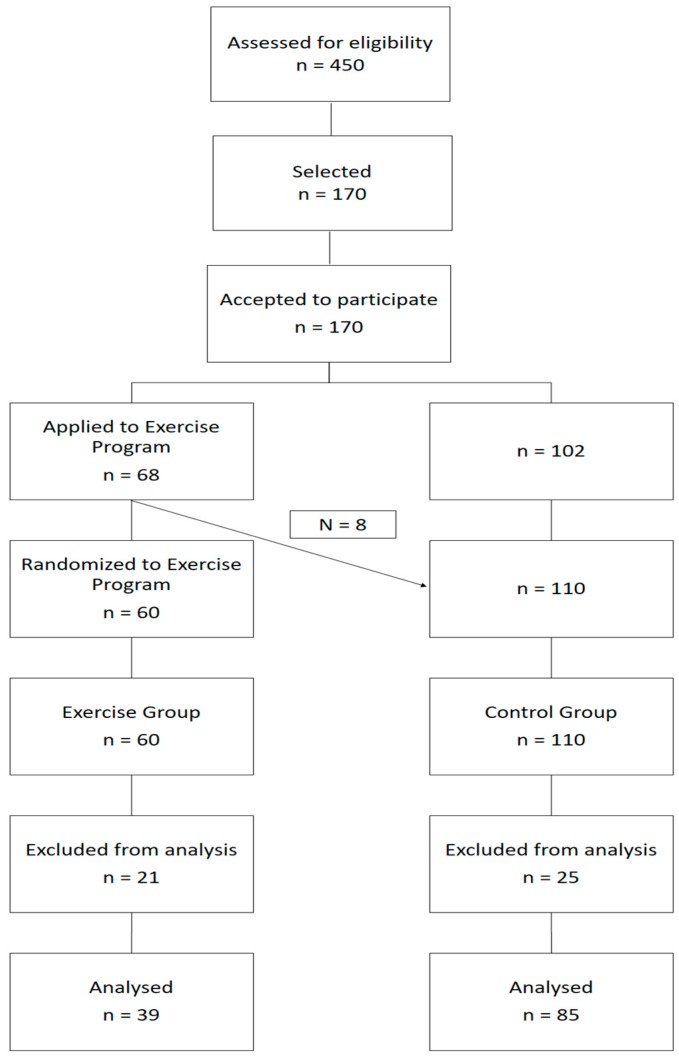
Sample diagram.

After exclusion criteria, final sample was composed of 124 Caucasian individuals (Table 1, Figure 2).

Adherence to the exercise program was 80.17 ± 10.28%. Thirteen adverse events were recorded during the course of the 108 exercise sessions: six symptomatic hypoglycemia (blood glucose < 72 mg/dL); four musculoskeletal injuries; and three non-specific indispositions. None of these events influenced the adherence results.

The exercise session’s overall intensity (score at the end of each session), assessed by Borg’s scale [6–20 points], was 13.45 ± 1.41 points.

Table 2 presents the mean values of the studied variables in the two evaluation moments.

A significant time * group interaction effect was identified for HbA1c (F = 18.025; *p* < 0.001; η^2^_p_ = 0.127), FPG (F = 17.559; *p* < 0.001; η^2^_p_ = 0.124), total cholesterol (F = 18.820; *p* < 0.001; η^2^_p_ = 0.132), LDL cholesterol (F = 16.770; *p* < 0.001; η^2^_p_ = 0.119), HDL cholesterol (F = 72.436; *p* < 0.001; η^2^_p_ = 0.369), and triglycerides (F = 82.555; *p* < 0.001; η^2^_p_ = 0.400), SBP (F = 69.704; *p* < 0.001; η^2^_p_ = 0.360), DBP (F = 53.479; *p* < 0.001; η^2^_p_ = 0.301), BMI (F = 150.683; *p* < 0.001; η^2^_p_ = 0.549), waist circumference (F = 168.498; *p* < 0.001; η^2^_p_ = 0.576), and the 10-year risk of CAD events (F = 24.676; *p* < 0.001; η^2^_p_ = 0.166). At baseline non-significant differences were observed between EXE and CON for the studied variables (HbA1c, *p* = 0.143; FPG, *p* = 0.430; total cholesterol, *p* = 0.618; LDL cholesterol, *p* = 0.895; HDL cholesterol, *p* = 0.311; triglycerides, *p* = 0918; SBP, *p* = 0.325; DBP, *p* = 0.642; BMI, *p* = 0.976; waist circumference, *p* = 0.383; and the 10-year risk of CAD events, *p* = 0.155).

No significant differences were identified in HPA (non-supervised) between CON and EXE at baseline [600.00 (965.25) vs. 735.00 (984.75); *p* = 0.505] or final [650.00 (971.50) vs. 773.00 (1010.00); *p* = 0.383] by Mann–Whitney U tests for independent samples (data not shown in tables).

## 4. Discussion

The main finding of this study is that an exercise intervention implemented in a community setting with high-applicability exercise strategies, developed with low-cost material resources, and taking advantage of the local sports infrastructures, resulted in significant health benefits for the participants with significant improvements in glycemic control, lipid profile, blood pressure, anthropometric profile, and the 10-year risk of CAD events, in comparison to a control group.

Cardiovascular diseases are the leading cause of death among patients with diabetes, and prevention should be based on the control of hyperglycemia and major cardiovascular risk factors such as high blood pressure, dyslipidemia, overweight and obesity, smoking habits, and physical inactivity [3,26,27].

An absolute decrease of 1% in HbA1c is associated with a reduction of 15 to 20% in major cardiovascular events [28]. In this study the investigators observed a reduction of 0.32% in HbA1c and 2.47% in the 10-year risk of CAD events (fatal and non-fatal) compared to the control group.

The exercise program also improved FPG by 5.95%, total cholesterol by 6.97%, LDL cholesterol by 10.78%, HDL cholesterol by 6.93%, triglycerides by 19.12 %, SBP by 7.65%, DBP by 6.20%, BMI by 3.94%, and waist circumference by 4.78%, compared to the control group.

These changes could represent important health gains in terms of the patients’ cardiovascular morbidity and mortality. Physical activity is associated with a reduction in cardiovascular mortality, as well as a reduction in all-cause mortality in patients with T2D. Previous studies suggest the existence of a dose-response in this relationship [4,5,29].

In the literature, there are several studies that examine the effects of a combined exercise program (aerobic and resistance) over a long period (≥16 weeks) on glycemic control and cardiovascular risk factors in patients with T2D. This type of exercise programs have been shown to induce significant benefits in HbA1c [30,31,32,33], FPG [30,32], total cholesterol [30,32], LDL cholesterol [30,32], HDL cholesterol [30,33] triglycerides [32,33], SBP [30,33], DBP [30], BMI [31,34], waist circumference [30,31,32], and the 10-year risk of CAD events [30] in comparison to a control group. It is noteworthy, however, that these studies used different exercise protocols with different programs’ duration, sessions’ length, weekly frequency, muscle mass elicited, and exercise intensity. Furthermore, these studies presented exercise interventions developed with exercise machines, such as ergometers for aerobic exercise (treadmills, stationary bikes, rowing machines, steppers, and ellipticals) and/or resistance machines for resistance exercise. Among the published literature, few studies [35,36] have developed combined exercise programs for people with T2D exclusively with low-cost exercise strategies and using different exercise protocols that combine free walking with resistance exercises performed with bodyweight, elastic bands, and free weights, as well as stretching exercises. However, the effects on glycemic control and cardiovascular risk factors were not consistent.

Aylin et al. [36] observed a significant improvement in the HbA1c levels of a group of patients with T2D (*n* = 18; 51.39 ± 2.02 years of age; HbA1c 7.7 ± 0.4 %; BMI 28.45 ± 0.95 kg/m^2^; polymedicated; exercise adherence of 96 %) in comparison to a control group after an exercise program of only eight weeks comprising free walking (60–79% maximum heart rate) two days a week, and resistance exercises performed with bodyweight and free weights on two other days. However, no changes were observed in FPG, lipid profile, or BMI.

Another study, Praet et al. [35] found no significant changes in HbA1c in a group of patients T2D (61 ± 9 years of age; HbA1c 7.2 ± 1.4%; BMI 32.1 ± 5.2 kg/m^2^; polymedicated; exercise adherence of 75 ± 16%) after a 12-month exercise program, consisting of free walking (75% maximum heart rate) and resistance exercises performed with elastic bands and bodyweight three days a week. These authors did not use a control group and final values were compared to baseline. The researchers found no significant changes in FPG, lipid profile, and BMI, but did see significant improvements in SBP and DBP.

Patients’ baseline characteristics and different exercise protocols may explain the different health outcomes.

An innovative feature of our exercise protocol was the inclusion of specific agility/balance exercises. These exercises are recommended for populations with a risk of falls, such as the elderly population [22,37], which is the age group with the highest prevalence of T2D [1]. Yet, falls are also a potential problem in individuals with diabetes, since these patients have a higher risk of falling when compared with the non-diabetic [38].

Another novelty of our study was the inclusion of different training methods in aerobic exercise strategies such as moderate-intensity continuous training and high-intensity interval training (HIIT). HIIT was applied in exercises like the relay-races, walking with an external load, and performing obstacle and stair circuits. Vigorous-intensity exercises are associated with higher benefits in glycemic control and cardiovascular risk factors in patients with T2D [39,40], and a HIIT program seems to induce greater benefits in HbA1c than a continuous training program of moderate intensity [41].

The pharmacological regimen (for T2D, hypertension, and dyslipidemias) used by the participants of this study did appear to be equivalent at baseline between EXE and CON. Patients with pharmacological changes (an increase in the dose of medications) during the study period (EXE, *n* = 4; CON, *n* = 13) were withdrawn from the final analysis, but they finished the exercise program. Another potential confounding variable that was controlled for was lifestyle-related physical activity. IPAQ was used to assess HPA (non-supervised), an international tool validated for the Portuguese population and widely used in patients with T2D [20,42].

Some studies controlled the dietary pattern by using a quantitative assessment of food intake, with an analysis of the nutrient content of foods and beverages [30,31]. In our study only qualitative changes and the usage of food supplements during the study period were analyzed by a dietitian, which can be considered a limitation. However, three individuals (EXE, *n* = 1; CON, *n* = 3) were withdrawn from final analysis due to changes in dietary pattern.

Improvements in the CON group’s outcomes (except anthropometric profile) were also observed. The authors interpret these changes as the result of the cumulative effects of the pharmacological treatment and also as the influence of participating in a study with regular evaluation of the pharmacological treatment’s compliance. These improvements highlight the importance of a control group in this type of lifestyle research.

The main limitation of this study was the lack of a complete randomization process between the EXE and CON. Since there were 60 vacancies available for the exercise program and only 68 applicants, there were not enough participants to have two randomized groups, and this study was classified as a quasi-experimental design. The authors are aware that this methodological approach was not ideal for the purpose of this study; however, it was the only possible design in real-world conditions.

Another limitation is the high number of dropouts and withdrawals due to low adherence (<65%) to the exercise sessions (21.6% of initial EXE). These factors reflect the difficulties of lifestyle intervention in this population and are similar to those reported by other studies in the same area [31,35].

The issue of motivation and the adherence to exercise in people with T2D has been of interest to researchers in recent years, due to the difficulties experienced and reported by most intervention studies—particularly long-term studies. Difficulties in time management, a lack of social and family support, a lack of frame of mind, cultural barriers, discouraging activities, discomfort caused by exercise, costs of transportations, and adverse weather conditions are some of the main reasons cited as barriers to exercise practice by patients with T2D [43].

Despite the above-mentioned limitations, our study is strengthened by the long-term intervention (nine months), the integration of four different types of exercise (aerobic, resistance, agility/balance, and flexibility) within each exercise session, the inclusion of HIIT methods for aerobic exercise, the exclusive use of low-cost and high-applicability exercise strategies, the existence of a control group, and an adequate final sample size in both experimental groups. Further research is needed to analyze the cost-effectiveness of this type of intervention in comparison with other traditional exercise programs and compare its effects in different community settings.

Low-cost exercise interventions, such as walking-based activities and resistance exercises performed with bodyweight and free weights, were effective in inducing significant benefits in glycemic control and major modifiable cardiovascular risk factors in middle-aged and older patients with T2D. This finding may be important in helping to fight the diabetes world pandemic. Health policies should promote these interventions in community settings, especially in primary health care and elderly institutions.

## 5. Conclusions

The results of this study showed that a long-term, community-based, combined exercise program developed with low-cost exercise strategies was effective in inducing significant benefits in glycemic control, lipid profile, blood pressure, anthropometric profile, and the 10-year risk of CAD events in middle-aged and older patients with T2D.

## Figures and Tables

**Table 1:** Participants’ characteristics and pharmacological regimen.

Variable	Total	Control Group	Exercise Group
Age (years)	63.29 ± 7.20	63.88 ± 7.62	62.05 ± 6.14
Diabetes duration (years)	10.88 ± 5.48	11.32 ± 5.26	9.98 ± 5.88
Number of individuals	*n* = 124	*n* = 85	*n* = 39
Female	*n* = 64	*n* = 44	*n* = 20
Male	*n* = 60	*n* = 41	*n* = 19
Oral antidiabetics	*n* = 115 (92.74%)	*n* = 79 (92.94%)	*n* = 36 (92.31%)
Insulin	*n* = 50 (40.32%)	*n* = 35 (41.18%)	*n* = 15 (38.46%)
Antihypertensives	*n* = 102 (82.26%)	*n* = 71 (83.53%)	*n* = 31 (79.49%)
Lipid lowering agents	*n* = 79 (63.71%)	*n* = 55 (64.71%)	*n* = 24 (61.54%)

**Table 2:** Average values (± standard deviation) of the studied variables in the two evaluation points in both the intervention and control group.

Variable	Control Group			Exercise Group			
	Baseline	Final	Δ	Baseline	Final	Δ	*p*
HbA1c (%)	7.95 ± 0.87	7.39 ± 0.92	−0.56	7.71 ± 0.85	6.83 ± 0.62	−0.88	<0.001
FPG (mg/dL)	152.09 ± 34.42	138.54 ± 34.90	−13.55	146.88 ± 35.07	125.05 ± 28.61	−21.83	<0.001
Total cholesterol (mg/dL)	180.92 ± 35.78	169.85 ± 36.04	−11.07	184.15 ± 29.94	160.05 ± 26.66	−24.10	<0.001
LDL cholesterol (mg/dL)	105.27 ± 34.87	94.11 ± 35.28	−11.16	106.09 ± 27.86	83.41 ± 23.90	−22.68	<0.001
HDL cholesterol (mg/dL)	48.87 ± 10.90	50.65 ± 10.92	1.78	51.15 ± 13.42	56.56 ± 14.08	5.41	<0.001
Triglycerides (mg/dL)	133.75 ± 40.49	125.36 ± 39.97	−8.39	134.54 ± 39.45	100.37 ± 28.52	−34.17	<0.001
SBP (mmHg)	136.42 ± 12.60	134.91 ± 13.81	−1.51	134.02 ± 13.09	122.29 ± 10.25	−11.73	<0.001
DBP (mmHg)	79.33 ± 9.74	79.07 ± 10.13	−0.26	78.49 ± 8.99	73.37 ± 6.55	−5.12	<0.001
BMI (kg/m^2^)	30.97 ± 4.73	31.60 ± 4.73	0.63	31.00 ± 5.17	30.41 ± 4.96	−0.59	<0.001
Waist circumference (cm)	107.82 ± 10.25	109.85 ± 9.99	2.03	105.94 ± 13.30	102.87 ± 12.59	−3.07	<0.001
10-year risk of CAD *(%)	19.29 ± 10.27	17.32 ± 9.54	−1.97	16.56 ± 9.46	12.12 ± 7.34	−4.44	<0.001

Δ: variation between baseline and final; *p*: level of statistical significance of the time * group interaction effect determined by Split-Plot analysis of variance with repeated measures; HbA1c: glycated hemoglobin; FPG: fasting plasma glucose; SBP: systolic blood pressure; DBP: diastolic blood pressure; BMI: body mass index; CAD: coronary artery disease; * calculated by United Kingdom Prospective Diabetes Study Risk Engine v2.0 [19].
